# The Systemic Alterations of Lipids, Alanine-Glucose Cycle and Inter-Organ Amino Acid Metabolism in Swine Model Confirms the Role of Liver in Early Phase of Septic Shock

**DOI:** 10.3389/fphys.2019.00011

**Published:** 2019-01-28

**Authors:** Manuela Ferrario, Laura Brunelli, Fuhong Su, Antoine Herpain, Roberta Pastorelli

**Affiliations:** ^1^Department of Electronics, Information and Bioengineering, Politecnico di Milano, Milan, Italy; ^2^Department of Environmental Health Sciences, Istituto di Ricerche Farmacologiche Mario Negri IRCCS, Milan, Italy; ^3^Experimental Laboratory of Intensive Care, Erasme Hospital, Université Libre de Bruxelles, Brussels, Belgium; ^4^Department of Intensive Care, Erasme Hospital, Université Libre de Bruxelles, Brussels, Belgium

**Keywords:** swine, septic shock, metabolomics, liver functionality, energy metabolism, lactate

## Abstract

Septic shock is a medical emergency and is one of the main causes of mortality in critically ill patients. Given the pathophysiological complexity of sepsis spectrum and progression in clinical settings, animal models become essential tools to improve patient care, and to understand key mechanisms that may remain masked from the heterogeneity of clinical practice. Our aim was to verify whether the metabolic constellations we previously reported for septic shock patients appear also in our septic shock swine model as systemic markers of early disturbances in energy metabolism and hepatic homeostasis. Septic shock was induced in anesthetized, instrumented, and ventilated adult swines by polymicrobial peritonitis. Hemodynamic and serial measurements of arterial and mixed venous blood gasses were made. Laboratory measurements and mass spectrometry-based targeted quantitative plasma metabolomics were performed in blood samples collected at baseline, at shock and at fully resuscitation after fluids and vasopressors administration. Data elaboration was performed by multilevel and multivariate analysis. Changes in hemodynamic, blood chemistry, and inflammatory markers were in line with a septic shock phenotype. Time course alteration of systemic metabolites were characterized by marked decreased in phosphatidylcholines and lysophosphatidylcholines species, altered alanine-glucose cycle and inter-organ amino acid metabolism, pointing toward an early hepatic impairment similarly to what we previously reported for septic shock. This is the first study in which an experimental swine model of septic shock recapitulates the main metabolic derangements reported in a clinical setting of shock. These events occur within hours from infections and may act as early metabolic features to assist in evaluating subclinical hepatic alterations and pave the way to improve the management of septic shock.

## Introduction

Septic shock is a life threatening condition that can develop subsequent to infection. It is a subset of sepsis with underlying circulatory and cellular/metabolic abnormalities associated with higher mortality rates (Angus et al., 2001; Singer et al., 2016). Septic shock causes progressive failure of vital homeostatic mechanisms culminating in immunosuppression, coagulopathy and microvascular dysfunction, which can lead to refractory hypotension, organ failure and death.

Early supportive therapy with fluid resuscitation and vasopressors to restore hemodynamics and reduce tissue hypoperfusion is decisive for the patient’s outcome and has figured in treatment guidelines for decades (Dellinger et al., 2013). However, mortality rate for septic shock may reach 40% even in the era of early recognition and treatment with today’s poor prognosis, mainly related to multi-organ dysfunction syndrome (MODS) (Rhee et al., 2017). The multiple organ involvement, which can lead to death within a few days, suggests that this concomitant process is systemic in nature and initiated in parallel with inflammatory response.

Given the pathophysiological complexity of sepsis spectrum and progression in clinical settings, animal models become essential tools to improve patient care, and to understand key mechanisms that may remain masked from the heterogeneity of clinical practice.

Several swine models of sepsis and septic shock have been developed to mimic the human disease progression and clinical settings by utilizing standard clinical treatments such as fluid resuscitation, antibiotics, and mechanical ventilation (Kubiak et al., 2011; Li et al., 2013; Revie et al., 2013; Corrêa et al., 2014; Chalkias et al., 2015; Vassal et al., 2015; Zhao et al., 2015; Duburcq et al., 2017). However, critiques on the relevance of experimental data regarding the translation to clinical sepsis/septic shock have been raised in the scientific community (Piper et al., 1996; Deitch, 1998; Esmon, 2004; Dyson and Singer, 2009; Opal and Patrozou, 2009). Aware of such criticism, our main goal in this study was to investigate whether our previous metabolic features characterizing septic shock patients (Ferrario et al., 2016; Cambiaghi et al., 2017) would have been observed also in a swine model of septic shock.

Previously, we found that lipids species alteration played an important role in individual patient responses to infection, where reduction in circulating lysophosphatidylcholines species, less change in plasmalogens concentration in combination with larger increment of alanine were associated with non-responsiveness to therapy (Cambiaghi et al., 2017). We speculated that alanine modulation indicated a possible alteration in the glucose-alanine cycle in the liver, providing a different picture of early liver functionality disturbances in the non-responder patients. Therefore, we aim to verify whether such metabolic constellations occurring in septic shock patients appear also in the septic shock swine model as systemic markers of early disturbances in energy metabolism and hepatic homeostasis, where liver acts not only as inflammatory barrier, but as organ actively involved in energy pathways.

In order to allow a robust comparison of the metabolomics data, we applied the same mass spectrometry-based target metabolomics strategy used for our previous clinical settings. Metabolomics analyses and biochemical/physiological measurements were conducted at three time points in septic shock and sham animals, i.e., at baseline, after shock development and after fully resuscitation, in order to capture changes in metabolic and physiologic trajectories.

## Materials and Methods

We performed a controlled experimental study on a large animal model of septic shock induced by a polymicrobial peritonitis on adult swine in our Experimental Laboratory of Intensive Care (LA1230336), in the Université Libre de Bruxelles.

The local animal ethics committee (Comité Ethique du Bien-Être Animal) approved the present study (protocol 641 N) and we followed the EU Directive 2010/63/EU for animal experiments and the ARRIVE guidelines for animal research.

### Study Design

Nine pigs (*Sus scrofa domesticus*) of both sex, aged 4 to 6 months, with a mean (± SD) weight of 42,3 ± 3,9 kg, were obtained from a local farm (BE 400108–48). Animals were fasted for 18 h prior to the start of the experiment with free access to water. This is a closed chest and low invasive instrumentation animal model with all the catheters and introducers inserted percutaneously under echographic guidance.

#### Anesthesia

The animals were sedated in their cage with an intramuscular injection of 1.5 mg/kg midazolam (Dormicum; Roche, Belgium) and 5 mg/kg azaperone (Stresnil, Eli Lilly Benelux, Belgium) in the neck. After transportation to the operating room, a 14 G peripheral venous line (14G, Surflo IV Catheter, Terumo Medical Company, Belgium) was placed into a vein of the ear to provide vascular access and a 4,5 Fr arterial catheter (Leader-Cath, Vygon, France) was placed in the left common femoral artery for invasive arterial pressure monitoring and blood samples collection. The animals underwent endotracheal intubation following induction of anesthesia with the intravenous injection of 3 μg/kg sufentanil (Sufenta Forte, Janssen-Cilag, Belgium), 1 mg/kg propofol (Propovet, Zoetis, Belgium) and 0.5 mg/kg of rocuronium (Esmeron, Organon, The Netherlands).

A central venous access for drugs infusion was obtained via a four lumen central venous line inserted inside the left femoral vein (Edwards Life Sciences, California, United States). General anesthesia and analgesia were achieved using continuous inhalation of sevoflurane 1,8 to 2,5 % MAC (Sevorane, Abbott, Belgium) and continuous infusion of sufentanil 1 to 4 μg/kg/h (adapted according to the response to painful stimulations, as a nasal septum pinching), in association with rocuronium continuous infusion to avoid shivering.

Mechanical ventilation was performed in volume-controlled mode (Primus, Draëger, Germany) with tidal volumes of 8 mL/kg and a PEEP set at 5 cm H_2_O. Arterial and venous blood gasses were analyzed live in the operating room (Cobas b-123, Roche, Switzerland) and the respiratory rate was adjusted to maintain PaCO_2_ values between 35 and 49 mmHg – aiming an arterial pH between 7,35 and 7,45 – while fraction of inspired oxygen (FiO_2_) was set at a value of 0,30 at baseline and adjusted to keep PaO_2_ > 90 mmHg. Fluid maintenance was provided by a balanced crystalloid (Plasmalyte, Baxter, Belgium) perfusion at 3 to 5 ml/kg/h, aiming a normal volemia at baseline, according to an arterial pulse pressure variation (PPV) ≤ 12% (and a negative fluid challenge in case of doubts, i.e., PPV remaining in the gray zone). Central temperature was maintained between 38,5 and 39,5°C with a warming blanket and hypoglycemia avoided by a continuous 10% glucose solution infusion (1 to 2 ml/kg/h).

#### Instrumentation

After a supra-pubic mini-laparotomy, a 14 Fr Foley catheter (Beiersdorf AG, Germany) was introduced into the bladder to monitor urine output (UO) and two large bore abdominal drains were placed in each side of the abdominal cavity for later introduction of the feces. A 7 Fr introducer was inserted into the right external jugular vein and a pulmonary artery catheter (CCO; Edwards LifeSciences, California, United States) was advanced in a pulmonary artery for continuous cardiac output (CO), right heart pressures and mixed venous oxygen saturation monitoring. Both femoral artery pressure and pulmonary artery pressure signals were continuously displayed (SC9000, Siemens, Germany) and exported to an A/D recording station (Notocord Hem, Notocord, France), similarly to EKG and cardiac output signals.

#### Experimental Protocol

After instrumentation, the animals were allowed to rest for approximately 2 h (premedication wash-out) after which the first baseline measurements and blood samples were taken (baseline, T1). The animals were then randomized for sepsis induction (shock group, *n* = 6) or a sham procedure (sham group, *n* = 3). Sepsis was induced by the intraperitoneal instillation, via the two abdominal drains, of 3 g/kg of autologous feces collected in the cage, filtered and diluted in 300 ml of glucose 10%, after which the drains were clamped. During septic shock onset, fluid maintenance was reduced to 1 ml/kg/h until the mean arterial pressure (MAP) has decreased below 50 mmHg owing to vasodilation and capillary leakage. Thereafter, fluid maintenance perfusion was moderately increased to avoid a further lethal decrease in blood pressure and to keep the animal alive for one more hour of severe hypotension (MAP goal between 45 and 50 mmHg), in order to consolidate the peripheral hypoperfusion and the multiple organ failure, after which the second time point T2, i.e., septic shock condition, was defined. Immediately after a series of hemodynamic measurements and blood samples, a full fluid resuscitation was initiated with both the same rate of the balanced crystalloid perfusion and an additional colloid perfusion (Geloplasma, Fresenius Kabi, France), aiming the same target as at baseline (PPV < 12% ± negative fluid challenge). The abdominal drains clamping was removed, to avoid fluid accumulation in the third space that otherwise would have led to an intra-abdominal hypertension. After 120 min of hemodynamic stabilization, defined by a stable MAP and no further increase in CO, additional hemodynamic measurements and blood samples were taken again (T3, first resuscitation). Finally, a vasopressor therapy was introduced, on top of the fluid resuscitation, with a continuous infusion of norepinephrine (Noradrenaline tartrate, Aguettant, Belgium) at a fixed dose of 0,3 μg/kg/min for one hour, after which the last series of hemodynamic measurements and blood samples were taken again (T4, full resuscitation). Animal were then euthanized with a potassium chloride injection and an overdose of thiopental.

In the septic shock group, two out of six animals were immediately fully resuscitated already with vasopressor because of the severity of the distributive shock, therefore they skipped the measurements at time point T3. For this reason, in the following we refer only to baseline (T1), shock (T2) and to fully resuscitation (T4) period.

#### Measurements

At each time point, arterial blood samples were collected and EDTA-plasma isolated for metabolomics and laboratory analyses after immediate refrigerated centrifugation. In addition, hemoglobin concentration, blood lactate and electrolyte concentrations were measured (Cobas b-123, Roche, Switzerland) on top of the arterial and mixed-venous blood gasses already mentioned.

### Metabolomics

Targeted quantitative approach using a combined direct flow-injection and liquid chromatography (LC) tandem mass spectrometry (MS/MS) assay (AbsoluteIDQ 180 kit, Biocrates, Innsbruck, Austria) was applied for the metabolomics analysis of plasma swine samples. The assay quantifies 186 metabolites from six analyte groups: acylcarnitines, amino acids, biogenic amines, hexoses (sum of hexoses), glycerophospholipids and sphingomyelins. The method combines derivatization and extraction of analytes with the selective mass-spectrometric detection using multiple reaction monitoring (MRM) pairs. Methodological details and data preprocessing have been extensively reported in our previous articles (Ferrario et al., 2016; Cambiaghi et al., 2017). Measurable metabolites and their extended names and abbreviations are listed in Supplementary Table S1.

### Statistical Analysis

We used the Wilcoxon signed-rank test to assess significant changes from baseline to shock and resuscitation time points within the same group. We adopted the Wilcoxon rank-sum test to verify significant differences in the parameters values between the two groups (sham and shock animals) separately at each time point. Significance was considered with a *p*-value <0.05.

### Multivariate and Multilevel Analyses of Metabolomics Data

We adopted the MultiLevel Simultaneous Component Analysis (MLSCA) and the multilevel partial least square discriminant analysis (MLPLSDA) model as proposed in De Noord and Theobald (2005) and Westerhuis et al. (2010). In the multilevel models the variation between subjects and the variation within subjects are separated.

The between subject variation in multilevel data analysis is performed on the average of the observations, whereas the within subject variation is performed on the net differences between paired observations. The initial step in multilevel model is therefore to separate the between subject variation from the within subject variation.

Total data matrix *X* (*N* × *J*) contains measurements for *I* subjects observed at *K_i_* time points on J variables, where total number of observations is $N=\sum_{i=1}^{I}K_{i}$. An element *x_ijk_i__* of matrix X, which contains a measurement of subject *i* on variable *j* at time point k_i_, can be decomposed as

(1) $$x_{ijk_{i}}=x_{.j.}+(x_{ij.}-x_{.j.})+(x_{ijk_{i}}-x_{ij.})$$

where *x*_.*j*._ is the overall mean for variable *j* and *x*_*ij*._ is the mean of subject *i* on variable *j*. The first term in Equation (1) is an offset that is constant across subjects and time points, the second term is a between-subject deviation, and the third term describes the within-subject deviation. Similarly, the sum of squares per variable can be separated in three parts, analogous to Analysis of Variance. The objective of these two-level model is to approximate the data, explaining the offset, between-subject, and within-subject variation as good as possible.

In the multilevel MLSCA model we assumed that the within –loadings matrices are the same for all the subjects. The MLSCA model for a subject *i* with data matrix *X_i_* (*K_i_* × *J*) containing the part of X with data from subject *i* (where *i* = 1,…, *I*) is the following:

(2) $$X_{i}=1_{K_{i}}m^{\prime }+1_{K_{i}}{t^{\prime }}_{b,i}{P^{\prime }}_{b}+T_{w,i}{P^{\prime }}_{w}+E_{i}$$

where 1_*K_i_*_ is a *K_i_* × 1 vector of ones, m (*J* × 1) contains the offsets of the J variables, *t*_*b,i*_ (*R_b_* × 1) contains the between-subject scores of subject *i* for *R_b_* retained between-components, *P_b_* (*J* × *R_b_*) is the between-subject loading matrix, *T_w,i_* (*K_i_* × *R_w_*) contains the within-subject scores for subject *i* for *R_w_* retained within-components, *P_w_* (*J* × *R_w_*) is the within-subject loadings matrix and *E_i_* (*K_i_* × *J*) contains the residual.

In the multilevel MLSCA the offset, the between-subject loadings, and the within-subject loadings are supposed equal for all subject, and the within-subject loadings are supposed to be time invariant as well. This means that the interpretation of the components is equal for all subject. The between-subject and within-subject variations in the data are described in separate terms, and are thus not confounded as in ordinary PCA. Given these hypotheses, the three parts of the model can be solved separately.

The between-subject part can be obtained from the matrices *X_b_* (*I* × *J*), which contain the mean vectors of the *I* subjects, by means of the singular value decomposition (SVD).

The within-subject part of the model can be estimated from the matrix *X_w_* (*N* × *J*), which consists of the concatenated centered matrices *Xi*, by means of the singular value decomposition.

In the multilevel partial least square discriminant analysis (PLS-DA), instead of using the simple SVD as in MLSCA, the projections are guided by the outcomes (Y matrix).

In these study, we performed a MLSCA model on *I* = 6 septic shock animals at the three time points (*K_i_* = 3) and we performed also two separate MLPLSDA: a model to analyze the within-subject variations occurring from baseline to shock and a second model to analyze the within-subject variations occurring from baseline to resuscitation. In such a way after the decomposition of the within- and between-subject variability a two class PLSDA is applied as the outcome consists in two distinct time point.

The models were analyzed on *J* = 17 parameters which are the metabolites under investigation (Glucogenic AA, Tot PC, LPC16+LPC18, Tot SM, Fisher ratio, PC ae C40:2, Alanine, Arginine, Citrulline, Glutamine, Glutamate, Ornithine, Tyrosine, ADMA, Creatinine, Glucose and Albumin).

In such a way, it is possible to understand in a multivariate fashion which species are most involved in determining the within-subject variation from baseline to shock condition and after resuscitation.

Finally, we applied a linear discriminant analysis (LDA) to two couples of specific molecular species (LPC and albumin, the ratio of alanine and glutamate and the ratio of glucose and lactate). Similarly to the MLSCA model, we considered *I* = 6 septic shock animals at the three time points (*K_i_* = 3) and we performed a three class LDA to the within-subject variability estimated from the matrix *X_w_* (*N* × *J*) where *J* = 2, i.e., the couple of species under investigation.

## Results

### Changes in Hemodynamic, Blood Chemistry Parameters and Inflammatory Markers in Septic Shock Swine

Table 1 shows the changes induced by septic shock at each time point as regard the key hemodynamic parameters, arterial and mixed-venous blood gasses, hemoglobin concentration, blood lactate, and electrolyte concentrations. The data are in line with the characteristic phenotype of septic shock. During the initial phases of sepsis, vasodilation and capillary leak caused filling pressures to be low and successively the fluid resuscitation produced the typical hyperdynamic state with increased cardiac output and decreased systemic vascular resistance. Serum bicarbonate decrease and blood lactate levels increased, although not reaching extremely high levels. As shock progressed, metabolic acidosis worsened, and blood pH decreased in spite of resuscitation.

**Table 1:** Values of hemodynamic parameters and laboratory analyses.

	Baseline	Shock	Resuscitation
HR (bpm)	78.5 (72.0, 91.0)	130.0 (101.0, 160.0)^∗^	147.0 (144.0, 152.0)^∗^
DAP rad (mmHg)	56.0 (54.0, 59.0)	36.0 (33.0, 37.0)^∗^	42.0 (35.0, 48.0)^∗§^
MAP rad (mmHg)	74.5 (71.0, 78.0)	46.0 (45.0, 46.0)^∗^	65.5 (55.0, 70.0)^#§^
CO ml/min	4950 (4300, 5400)	3000 (2500, 4275)	9350 (8000, 9900)^∗∘^
SvO_2_ %	65.0 (61.0, 69.0)	59.5 (53.0, 68.0)	75.0 (68.0, 78.0)
Lactate mmol/L	0.900 (0.900, 0.900)	1.700 (1.300, 2.100)^∗^	1.900 (1.750, 3.175)^∗∘^
T°C	38.9 (38.8, 39.1)	38.4 (37.7, 39.2)	38.8 (38.2, 39.1)
pH	7.480 (7.470, 7.490)	7.415 (7.410, 7.440)^∗^	7.435 (7.410, 7.450)^∗^
PaCO_2_ mmHg	46.2 (45.2, 48.1)	48.8 (47.0, 49.8)	48.3 (47.6, 49.1)
PaO_2_ mmHg	127 (124, 133)	138 (133, 154)	99 (70, 157)
HCO_3_- mmol/L	33.1 (33.0, 35.6)	31.1 (30.7, 31.2)^∗^	30.7 (30.0, 31.8)^∗^
BE	8.850 (8.400, 11.000)	5.850 (5.200, 5.900)^∗^	5.650 (4.600, 7.000)^∗^
Sat O_2_ %	100.0 (99.0, 100.0)	100.0 (99.0, 100.0)	98.0 (89.0, 99.0)
Hct %	27.0 (25.0, 28.4)	33.5 (28.0, 37.0)	25.6 (24.0, 30.0)°
Na^+^ mmol/L	132 (131, 134)	131 (131, 134)	134 (133, 135)
K^+^ mmol/L	4.0 (4.0, 4.3)	3.8 (3.6, 4.3)	4.4 (4.2, 4.6)°
Cl^-^ mmol/L	99.5 (97.0, 101.0)	98.0 (96.0, 99.0)^#^	97.5 (97.0, 99.0)
anion gap	-1.0 (-1.0, -0.6)	3.9 (1.3, 5.0)^∗^	6.0 (2.0, 6.2)^∗^
Ca^++^ mmol/L	1.235 (1.200, 1.260)	1.210 (1.180, 1.270)	1.125 (1.080, 1.170)^∗§^
Glucose mmol/L	93.0 (86.0, 99.0)	69.0 (62.0, 82.0)^∗^	67.0 (60.0, 86.0)


*Values of hemodynamic parameters, arterial and mixed-venous blood gasses, hemoglobin concentration, blood lactate, and electrolyte concentrations in shock group animals. Comparisons with baseline values: ^∗^Wilcoxon sign-rank test *p* < 0.05, ^#^Wilcoxon sign-rank test *p* = 0.063. Comparisons with shock values: ^§^ Wilcoxon sign-rank test *p* < 0.05, ^° ^Wilcoxon sign-rank test *p* = 0.063. Values are reported as median (25th, 75th) percentile; HR, heart rate; DAP rad, diastolic arterial pressure from radial artery; MAP rad, mean arterial pressure from radial artery; CO, cardiac output; T, body temperature; PaCO_*2*_, arterial carbon dioxide partial pressure: PaO_*2*_, arterial oxygen partial pressure; Sat O_*2*_, oxygen content; BE, base excess; Hct, hematocrit; anion gap measured as AG = Na^+^ – (Cl^-^ + HCO_3_^-^).*

**FIGURE 1:**
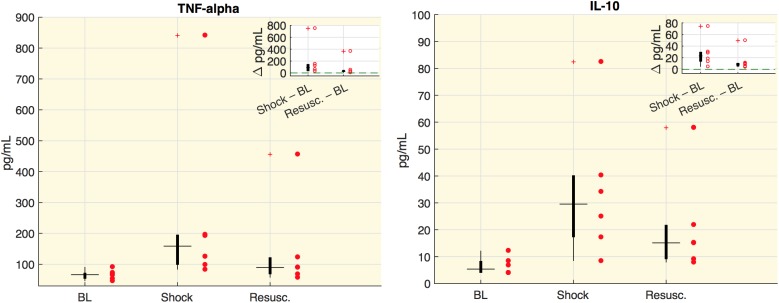
Cytokine plasma concentration. Boxplot of cytokine plasma concentration (pg/ml). Each circle represents the value of an animal. In the corner of each figure, boxplots of delta values are shown, i.e., the differences between shock and baseline values (BL) and the differences between the values after resuscitation (Resusc.) and baseline values. All the variations with respect to baseline were significant (Wilcoxon sign-rank test *p* < 0.05).

Values of cytokines at each time point are shown in Figure 1. In particular, we measured the tumor necrosis factor alpha (TNF-alpha), which is involved in systemic inflammation particularly during the acute phase reaction, and the interleukin 10 (IL-10), which is an anti-inflammatory cytokine capable to inhibit the synthesis of pro-inflammatory cytokines, such as TNF-alpha. As expected, both cytokine concentrations significantly increased and remained high after the shock was induced.

### Time Course Alteration of Systemic Metabolic Features in Septic Shock Swine

**FIGURE 2:**
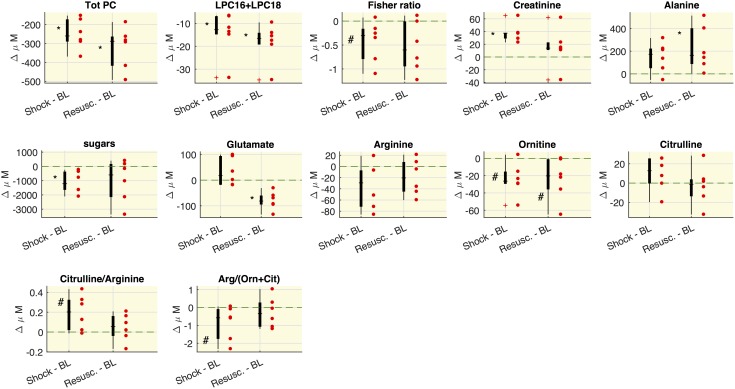
Variations of metabolites concentrations. Boxplot of delta values, i.e., the differences between shock and BL and the differences between the values after the resuscitation (Resusc.) and baseline values. Each circle refers to an animal. The symbols highlight the significant differences with respect to baseline values: ^∗^Wilcoxon sign-rank test *p* < 0.05, ^#^Wilcoxon sign-rank test *p* = 0.063. The green shaded line marks Δ = 0. LPC16+LPC18 represents the sum of LPC species C16:0+16:1+18:0+18:1+18:2.

The selected metabolite concentrations (μM) at each time point are reported in Table 2, while Figure 2 shows the delta (Δ) values of metabolites concentrations, as differences between shock vs baseline and between resuscitation vs baseline. In particular, we observed in shock a significant marked decrease of the overall amount of phosphatidylcholines species (total PC), of the sum of palmitoyl (C16) and stearoyl (C18) lysophosphatidylcholines species (LPC) at different saturation (C16:0+16:1+18:0+18:1+18:2) expressed as LPC16+LPC18, the plasmenylcholine PCaeC40:2, the overall amount of sphingomyelins (Total SM). A decrease trend in ornithine and the citrulline/glutamine ratio was also observed. As illustrated also in Figure 2, these metabolic features remained reduced even after resuscitation. From baseline to shock, the global arginine bioavailability ratio, i.e., arginine/(ornithine+citrulline) and the Fisher ratio decreased. On the contrary, the citrulline/arginine ratio increased from baseline to shock. Glutamine significantly increased from baseline to shock and remained elevated after resuscitation.

**Table 2:** Values of plasma metabolites concentration and metabolite ratios in the shock group.

	Baseline	Shock	Resuscitation
Glucogenic AA	3365 (2949, 3599)	3469 (3407, 3773)^#^	3771 (3576, 4419)
Tot LPC/PC	0.052 (0.044, 0.058)	0.058 (0.044, 0.074)	0.045 (0.042, 0.047)
Tot PC	452 (339, 529)	136 (118, 247)**^∗^**	77 (36, 115)^**∗**§^
LPC16 + LPC18	20.356 (15.187, 20.595)	7.769 (6.119, 8.650)**^∗^**	3.253 (1.242, 4.700)^**∗**§^
Tot SM	30.970 (30.248, 31.799)	18.363 (15.395, 20.255)**^∗^**	7.227 (5.381, 8.221)^**∗**§^
Fisher ratio	2.953 (2.865, 3.560)	2.841 (2.515, 3.201)^#^	2.790 (2.514, 3.073)
PC ae C40:2	0.269 (0.255, 0.271)	0.149 (0.134, 0.163)**^∗^**	0.076 (0.061, 0.095)^**∗**§^
Creatinine	124 (122, 129)	164 (150, 180)**^∗^**	140 (123, 147)
Hexoses	5370 (4717, 6013)	4276 (4012, 4351)**^∗^**	4787 (3508, 5116)
Alanine	584 (467, 690)	711 (637, 782)	761 (651, 923)**^∗^**
Glutamate	202 (152, 249)	254 (223, 283)	138 (102, 172)^**∗**§^
Arginine	162 (142, 195)	133 (109, 139)	142 (135, 158)
Citrulline	60.300 (47.900, 66.800)	73.300 (67.000, 84.800)	62.700 (51.800, 75.900)
Ornitine	78.950 (70.700, 81.000)	58.450 (51.500, 64.600)^#^	59.000 (48.600, 78.900)^#^
Arg/(Orn+Cit)	2.409 (1.969, 3.062)	1.843 (1.306, 2.027)^#^	2.339 (1.652, 2.993)
Glutamine	520 (452, 638)	750 (628, 828)**^∗^**	910 (784, 1100)**^∗^**°
ADMA	1.325 (1.160, 1.900)	1.485 (1.310, 1.560)	1.285 (1.260, 1.480)
Tyrosine	75.850 (68.100, 84.000)	59.400 (45.600, 69.700)	44.500 (41.100, 48.400)**^∗^**°
Citrulline/Glutamine	0.122 (0.088, 0.144)	0.106 (0.083, 0.127)^#^	0.076 (0.067, 0.080)^**∗**§^
Citrulline/Arginine	0.407 (0.319, 0.496)	0.534 (0.482, 0.751)^#^	0.436 (0.328, 0.592)
Ornitine/Arginine	0.0087 (0.0074, 0.0109)	0.0106 (0.0092, 0.0144)	0.0093 (0.0088, 0.0110)
Ornitine/Citrulline	0.024 (0.017, 0.028)	0.018 (0.017, 0.020)	0.021 (0.017, 0.028)
ADMA/Arginine	0.492 (0.459, 0.544)	0.432 (0.387, 0.529)	0.424 (0.377, 0.458)**^∗^**


*Concentrations (μM) and ratios are reported at each time point as median, 25th and 75th percentiles. Fisher ratio is the ratio between the branched-chain amino acids (leucine, valine, isoleucine) to aromatic aminoacids (phenylalanine, tyrosine). LPC16+LPC18 represents the sum of LPC species C16:0+16:1+18:0+18:1+18:2. ADMA, asymmetric dimethylarginine. Comparisons with baseline values: ^∗^Wilcoxon sign-rank test *p* < 0.05, ^#^Wilcoxon sign-rank test *p* = 0.063. Comparisons with shock values: ^§^Wilcoxon sign-rank test *p* < 0.05, ^° ^Wilcoxon sign-rank test *p* = 0.063.*

As regard the glucose-alanine cycle, we observed that alanine was significantly higher at resuscitation with respect the baseline values, glutamate significantly decreased after the resuscitation with respect to baseline and shock values and hexoses were significantly lower in shock with respect to baseline.

### Liver Functionality Markers in Septic Shock Swine

**FIGURE 3:**
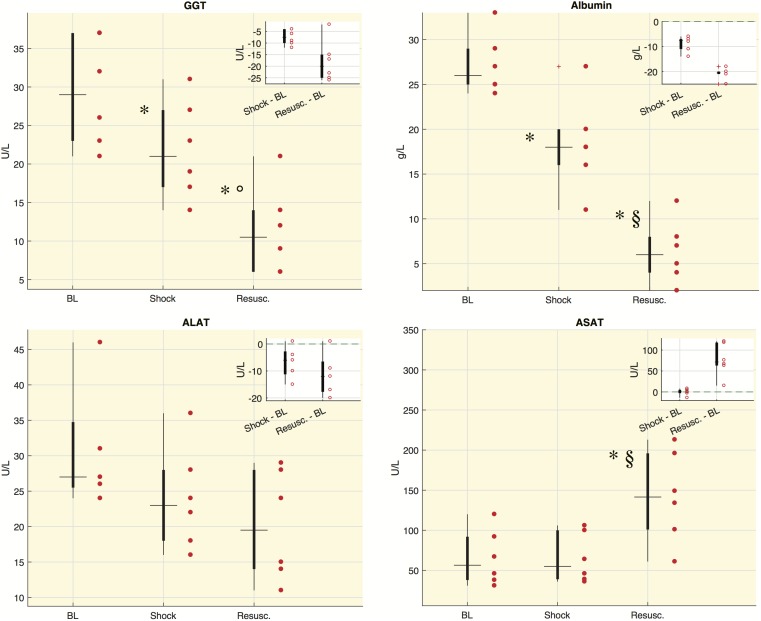
Laboratory values for each time point in shock group. Boxplot of laboratory values: each circle refers to an animal. In the corner of each figure, boxplots of delta values are shown, i.e., the differences between shock and BL and the differences between the values after resuscitation (Resusc.) and baseline values. The symbols highlight the significant difference with respect to baseline values: ^∗^Wilcoxon sign-rank test *p* < 0.05, and with respect to shock values: ^§^Wilcoxon sign-rank test *p* < 0.05, °Wilcoxon sign-rank test *p* = 0.063. GGT, gamma-glutamyltransferase; ALAT, alanine aminotransaminase; ASAT, aspartate aminotransferase.

Figure 3 shows the values distribution of blood analyses related to liver functionality. The concentration of albumin and gamma-glutamyltransferase (GGT) significantly decreased at either shock and resuscitation time points, whereas the concentration of aspartate aminotransferase (ASAT) increased significantly at the last time point. Alanine aminotransferase (ALAT) showed decreased concentrations from baseline, although the downward trend did not reach statistical significance. We computed also the ASAT/ALAT ratio. We found a significant increase from baseline to shock and in particular after the resuscitation [BL: 2.58 (1.56, 2.81), Shock: 2.89 (2.17, 2.94), Resusc.: 6.75 (6.21, 7.61)].

### Metabolic Features Association in Septic Shock Swine

**FIGURE 4:**
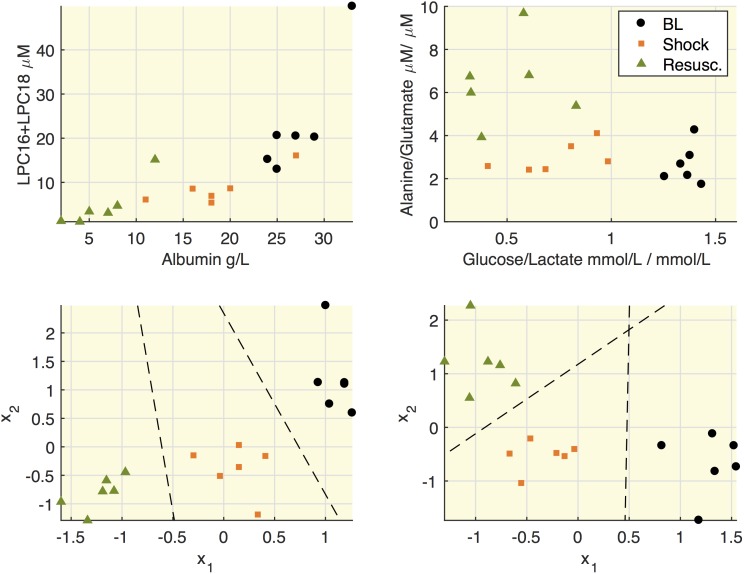
Relationships between lipids and albumin and between alanine and glucose. Upper panels: representation of concentrations of LPC16+LPC18 as sum of species C16:0+16:1+18:0+18:1+18:2 and albumin; the ratio between alanine and glutamate and the ratio between glucose and lactate. Each mark refers to an animal at the three time points (baseline, BL; shock; resuscitation, Resusc.). Lower panels represent the results of linear discriminant analysis (LDA) on the within-subject variability on the couples of species illustrated in the upper panel. *x*_1_ and *x*_2_ refers to the variation of each variable with respect to the subject mean and the lines mark the boundary obtained from LDA.

All the quantified metabolites are reported in Supplementary Table S2. Figure 4 highlights some associations between molecular species in septic shock model. We reasoned that since hypoalbuminemia was observed in our septic shock model and that albumin is a fatty acid transporter, it would have been plausible to find an association between these molecular species. Indeed, the upper panels show that both albumin and the sum of LPC16+LPC18 species decreased after shock. Similarly, when we focused on energy metabolic pathways, the glucose/lactate ratio decreased after shock passing from values higher than 1 at baseline to lower values than 1. This trend was paralleled by increased alanine/glutamate ratio particularly after the resuscitation. To note that the results of linear discriminant analysis (LDA) on the within-subject variability (lower panels) allowed the separation of each experimental phase from the others.

### Multilevel and Multivariate Analysis of Metabolomics Data in Septic Shock Swines

**FIGURE 5:**
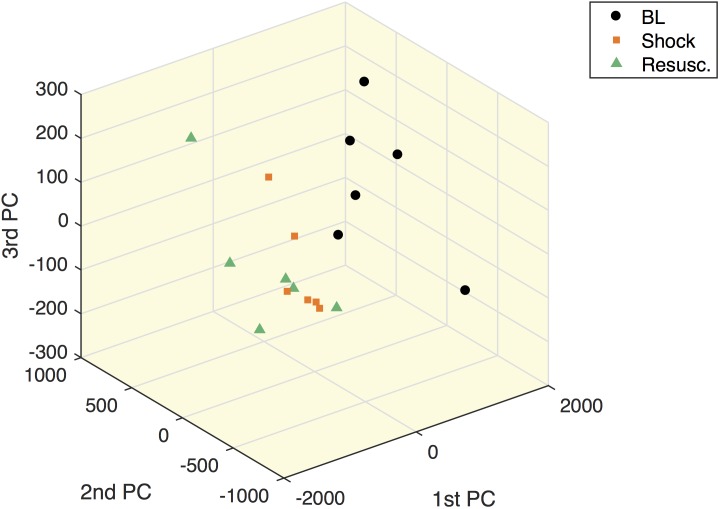
Multilevel simultaneous component analysis of metabolites concentrations in shock animals. The first three principal components of the within-subject part of the MLSCA which are related to intra-subject changes among the different time points.

Figure 5 shows the within-subject variation obtained from the MLSCA analysis. The baseline condition appears clearly separated from the other two time points.

Table 3 illustrates the VIP scores of the MLPLSDA models. We can observe that the sum of LPC16+LPC18 species, phosphatidylcholines (PC) and sphingomyelins (SM) are ranked in the first positions of VIP score in both the two models, so that they can be considered important drivers of the within-subject variations occurring from baseline to shock and to resuscitation conditions. The bioavailability of arginine seems to have a greater role in the first model related to within-subject variations occurring from baseline to shock, as already reported in the univariate analysis. The VIP score of glutamate, which is a substrate in the gluconeogenesis from alanine, is higher than 1 in the second model only, whereas creatinine has less importance in the second model with respect to the first one.

**Table 3:** Metabolites VIP scores.

MLPLSDA BL to shock		MLPLSDA BL to resuscitation	
	
	VIP		VIP
PC ae C40:2	1.346	Tot SM	1.394
Tot PC	1.334	PC ae C40:2	1.386
Creatinine	1.316	Tot PC	1.346
Tot SM	1.316	LPC16+LPC18	1.319
LPC16+LPC18	1.234	Tyrosine	1.315
Hexoses	1.179	Glutamate	1.310
Alanine	1.161	Glutamine	1.231
Glutamine	1.141	Citrulline/Glutamine	1.184
Ornitine	1.134	Fisher ratio	1.134
Citrulline/Arginine	1.091	Alanine	1.101
Fisher ratio	1.066	Ornitine	1.059
Arg/(Orn+Cit)	1.028	ADMA/Arginine	1.057
Arginine	0.962	Tot LPC/PC	0.899
Tyrosine	0.957	Glucogenic AA	0.887
Citrulline/Glutamine	0.922	Hexoses	0.865
Ornitine/Citrulline	0.795	Arginine	0.824
Glutamate	0.757	Creatinine	0.705
Citrulline	0.719	Citrulline/Arginine	0.571
Ornitine/Arginine	0.633	Arg/(Orn+Cit)	0.558
Tot LPC/PC	0.534	Ornitine/Arginine	0.402
ADMA/Arginine	0.526	Ornitine/Citrulline	0.249
Glucogenic AA	0.487	ADMA	0.243
ADMA	0.308	Citrulline	0.216


*Metabolites VIP scores of the two MLPLSDA models which represent the within-subject variations from baseline to shock and from baseline to resuscitation. LPC16 + LPC18 represents the sum of LPC species C16:0+16:1+18:0+18:1+18:2.*

### Metabolic Features Comparison Between Septic Shock and Sham Swine

**FIGURE 6:**
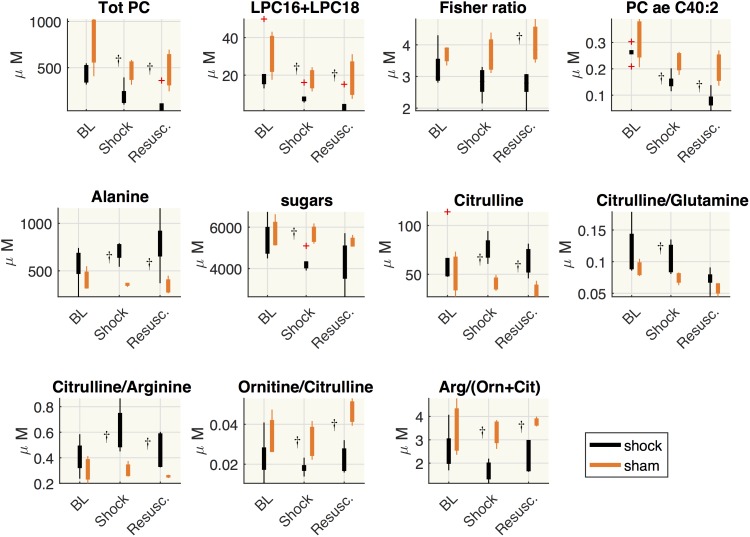
Comparisons between shock and sham animals. Boxplot of metabolites concentration at each time point: baseline (BL), shock and resuscitation (Resusc.). The symbol ^†^highlights the significant differences between shock and sham group (Wilcoxon rank sum test *p* < 0.05).

Figure 6 shows the distribution of the metabolite concentrations in shock and sham groups. In both shock and resuscitation period the overall amount of phosphatidylcholines species (total PC), the sum of LPC16+LPC18 species, the plasmenylcholine PCaeC40:2, the ornithine/citrulline ratio, the global arginine bioavailability ratio (GABR: arginine/ornithine+citrulline), were significantly lower in shock group with respect to sham animals, whereas alanine and citrulline/arginine were significantly higher. Hexoses and citrulline/glutamine were significantly enhanced during shock period only, whereas the Fisher ratio was significantly reduced after resuscitation in shock group with respect to sham animals.

## Discussion

Since the etiology and pathophysiology of septic shock are complex, it is difficult to develop a clinically accurate animal model of septic shock with relevance as translational model of sepsis-induced shock. Nevertheless, our swine model replicates the hemodynamic dysfunctions and the systemic inflammation that are typically found in clinical setting, although it does not reproduce the comorbidities that may complicate the clinical course of human septic shock.

Importantly, our model displays an altered metabolic landscape pointing toward an early hepatic impairment similarly to what we previously reported for septic shock patients (Cambiaghi et al., 2017). We confirmed here that the time course of metabolome changes in septic shock swines are mainly characterized by reduced plasma phospholipids species trajectories, altered alanine-glucose cycle and deranged inter-organ amino acid metabolism, all metabolic traits reported in septic shock patients (Bruins et al., 2003; Murch et al., 2006; Kao et al., 2009; Ferrario et al., 2016; Ahn et al., 2017; Cambiaghi et al., 2017).

To our knowledge, this is the first study in which an experimental model of septic shock recapitulates the main metabolic derangements reported in a clinical setting of septic shock.

Shock swines are characterized by reduced levels of PC, SM and of a cluster of LPC species composed by C16:0-, C16:1-, C18:0-, C18:1-, and C18:2-LPC. Further, this metabolites pool is lower in shock animals with respect to sham animals in the corresponding period of shock and after resuscitation.

LPCs have a very complex metabolism and play key roles in inflammatory responses (Lin et al., 2005; Schmitz and Ruebsaamen, 2010). Interestingly LPC species enhance the suppressive function of CD4+ CD25+ regulatory T cells (T_regs_) involved in anti-inflammatory signaling (Hasegawa et al., 2011; Osborn and Olefsky, 2012). Defects in T_reg_ function are considered to play a role in hepatic inflammation (Speletas et al., 2011; Peiseler et al., 2012) and low levels of LPC16 and 18 species were reported in inflammatory liver disease (Tanaka et al., 2012; Lehmann et al., 2013; Maricic et al., 2014). These observations suggest that decreased circulating LPC might hamper their anti-inflammatory effects and conceivably, may promote an excessive immune response.

Because the liver is known to be a major source of circulating PC and LPC (Sekas et al., 1985; Baisted et al., 1988), it is plausible their plasma levels might be influenced by the functionality of hepatic lipid-modifying enzymes, whose expression can be regulated by pro-inflammatory cytokines such as TNF-α (Tanaka et al., 2012). It is likely that the production of pro-inflammatory cytokines devoted to regulate the hepatic response in sepsis may further injure hepatocytes.

Importantly, we found the same imbalance of LPC/PCs cycling observed in non-responsive septic shock patients (Cambiaghi et al., 2017), therefore strongly supporting that hepatic homeostasis is very early compromised. Such lipid levels might act as metabolic messengers in signaling early liver suffering (e.g., energy status) with consequent implication in the host response (Liu et al., 2014).

To note that both albumin and the observed LPC cluster decreased after shock in our model. Hypoalbuminemia is frequently observed during acute disease state, as albumin is a negative acute-phase protein. Hypoalbuminemia may be caused by the combination of increased transcapillary escape ratio, accelerated catabolism and impaired hepatic synthesis (Nicholson et al., 2000; Gatta et al., 2012). Albumin acts as fatty acid transporter (van der Vusse, 2009) and its reduction may play a critical role in the lipid economy of the body (Novak et al., 2017).

Therefore, our findings might suggest that the concomitant drop in albumin and the reduction in LPC species may represent a vicious cycle with detrimental effect on the intensity of inflammatory responses in the liver.

The plausible impairment in the early functionality of the liver in septic shock swine is also supported by the derangement of many interconnected energy-related metabolic pathways. The enhanced circulating alanine indicates an alteration in the glucose-alanine cycle as we previously observed in non-responsive septic shock patients (Cambiaghi et al., 2017). The higher plasma alanine in shock animals may be a sign of lower hepatic capacity for conversion of alanine to glucose, whose enhanced elaboration by the liver (hepatic gluconeogenesis) is a prominent feature of the solid organ response to injury and provides fuel to the cellular elements of the inflammatory response. The glucose/lactate ratio decreased after shock, indicating an imbalance between glycolysis and gluconeogenesis. This trend paralleled the increased alanine/glutamate ratio, particularly after the resuscitation. The alanine/glutamate ratio represents the alanine bioavailability from the impaired hepatic glucose-alanine cycle as part of the gluconeogenesis pathway. In fact, ALAT catalyzes the transfer of an amino group from alanine to α-ketoglutarate, to produce glutamate and pyruvate, which enters the gluconeogenesis and is used for glucose synthesis. Interestingly, we found a downward trend of ALAT in shocked and resuscitated animals, although not statistically significant.

As regards the transaminases trends, we may refer to subclinical hepatic alterations and not an acute liver dysfunction. The observational window after the insult is too short to have an hypoxic hepatitis, which is related to hypoperfusion in the presence of hypovolemia as well as inadequate cardiac output. Moreover, the increased levels of both ALAT and ASAT are observed in animal model of sepsis for longer time windows such as 24 h after the insult (Idrovo et al., 2015). However, in McGill (2016) the author reviews the limitation of using ALAT and ASAT for monitoring liver function. It is generally thought that aminotransferase elevations are due to cell damage with plasma membrane disruption. Apoptotic cell death can be thought of as cell implosion; a controlled process with minimal release of proteins into the extracellular space. However, oncotic necrosis, which can be thought of as cell explosion, can occur in some cells secondarily after induction of apoptosis. The hallmarks of apoptosis (e.g., cell shrinkage, chromatin condensation, apoptotic body formation) are clearly observable in the liver in hepatocyte apoptosis models during the early phase of injury when there is no significant elevation in serum ALAT (McGill, 2016).

Disturbance in metabolism is one of the most critical feature in sepsis/septic shock as response of the body to mobilize nutritional resources for organs and immune cells (Bruins et al., 2000, 2003; Whelan et al., 2014). In this complex and dynamic scenario, the time-course increase of plasma alanine and glutamine, major sources of nitrogen and carbon in inter-organ amino acid metabolism, may reflect a reduced rate in their utilization by organs in septic shock swine. The rise of circulating glutamine may support dysregulation in glutamine transport and extraction system, either suggesting reduced utilization by the gut and/or impaired hepatic transformation into urea cycle. Indeed, intermediates of the urea cycle were also disturbed in shock animals, where the global arginine bioavailability ratio (GABR: arginine/ornithine+citrulline) was significantly reduced compared to sham animals. GABR reduction may reduce NO, vasodilator essential to vascular homeostasis, and may lead to endothelial dysfunction (Kao et al., 2009).

The liver receives compounds absorbed or released by the gut through the portal vein before they gain access to the systemic circulation. The raised plasma level of glutamate, citrulline, arginine, seen in our septic shock swine model, are likely due to dysregulation of the gut-liver axis conversion of glutamine to citrulline and arginine. The gut accounts for the major part of this conversion, although splanchnic citrulline release to the systemic circulation may be limited by hepatic citrulline extraction (Van De Poll et al., 2007; Marini, 2016). To note that both arginine and citrulline plays an essential role in the immune response to sepsis and a disturbed arginine-nitric oxide (NO) metabolism is associated with deteriorating organ functions (Wijnands et al., 2012, 2015). Taken together our data are consistent with recent studies revealing liver dysfunction as an early event in sepsis (Nesseler et al., 2012; Recknagel et al., 2012; Wang et al., 2014).

## Conclusion

The animal experimental data presented herein provide evidence that reduced plasma LPC/PC species trajectories, altered alanine-glucose cycle and deranged inter-organ amino acid exchange metabolism are early events in septic shock swine. These events occur within hours from infection and point to alterations in energy substrate utilization and hepatic biochemical perturbations. They may act as early metabolic features to assist in evaluating subclinical hepatic alterations. Being consistent with the metabolomics patterns differentiating non-responsive from responsive septic shock patients (Cambiaghi et al., 2017), such metabolic features may even represent therapeutic targets and therefore pave the way to improve the management of septic shock.

## Data Availability

Metabolomics data are in the Supplementary Material; other experimental data are available upon request.

## Author Contributions

MF conceived and designed the study, did the statistical analysis, contributed to data interpretation, drafted, revised, and approved the manuscript. LB did the metabolomics analysis, contributed to data interpretation, revised, and approved the manuscript. FS and AH designed and developed the animal model, clinical animal data acquisition and interpretation, revised, and approved the manuscript. RP conceived the study, coordinated metabolomics analysis and data interpretation, drafted, and approved the manuscript.

## Conflict of Interest Statement

The authors declare that the research was conducted in the absence of any commercial or financial relationships that could be construed as a potential conflict of interest.

## Abbreviations

ALAT: alanine aminotransferase
ASAT: aspartate aminotransferase
FiO_2_: fraction of inspired oxygen
GGT: gamma-glutamyltransferase
LC: liquid chromatography
LDA: linear discriminant analysis
LPC: lysophospatidylcholine
LPC16+LPC18: the sum of palmitoyl (C16) and stearoyl (C18) lysophosphatidylcholines species at different saturation (C16:0+16:1+18:0+18:1+18:2)
MAP: mean arterial pressure
MLPLSDA: MultiLevel partial least square discriminant analysis
MLSCA: MultiLevel simultaneous component analysis
MODS: multi-organ dysfunction syndrome
MRM: multiple reaction monitoring
MS/MS: tandem mass spectrometry
PC: phosphatidylcholine
PPV: arterial pulse pressure variation
SM: sphingomyelin
